# Supplementary material for: What does it take to learn the rules of RNA base pairing? A lot less than you may think
Source: bioRxiv. 2025 Aug 2:2025.07.31.668042. Preprint. [Version 1] doi: 10.1101/2025.07.31.668042 (PMC12324431; doi:10.1101/2025.07.31.668042)
Supplement: Supplement 1 [file media-1.gz › supplemental_material/experiments/g6_optimize_param_conus_rnabench_RNaseP_conus_rnabench_srp_random/losses_i89.pdf]

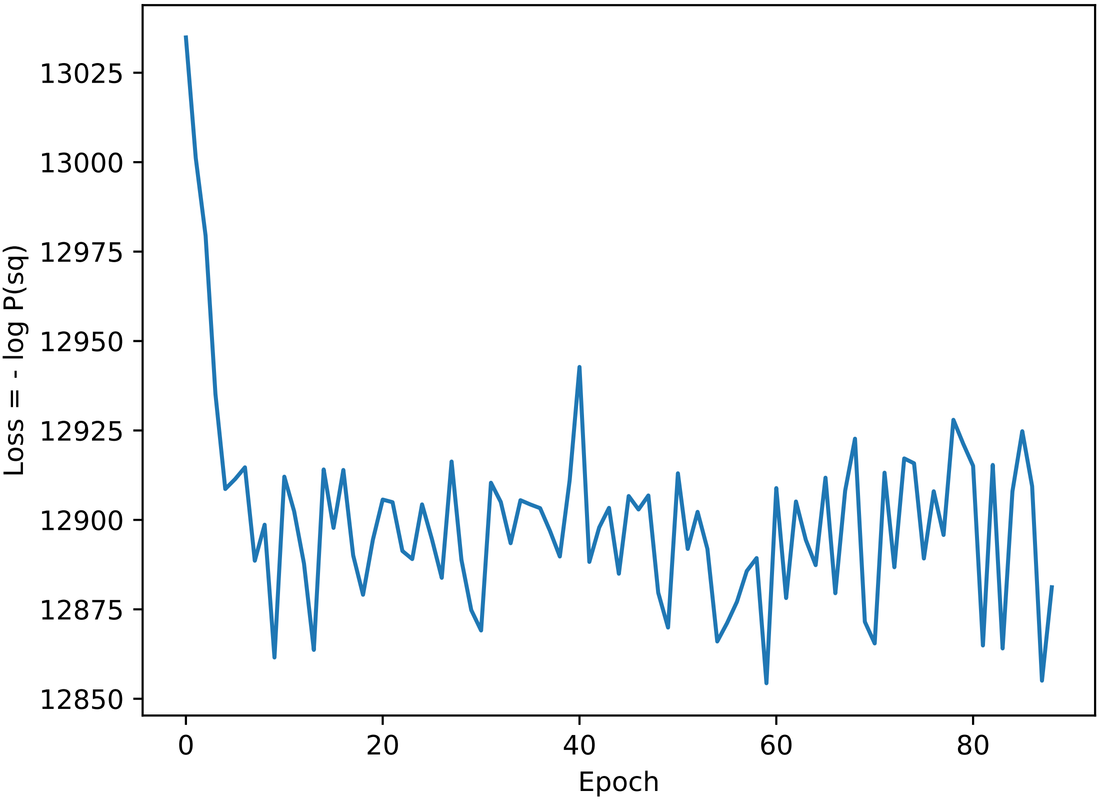

13025
13000
12975
12950
12925
12900
12875
12850
Loss = - log P(sq)
0
20
40
60
80
Epoch
